# Cyanidin-3-*O*-Glucoside Supplement Improves Sperm Quality and Spermatogenesis in a Mice Model of Ulcerative Colitis

**DOI:** 10.3390/nu14050984

**Published:** 2022-02-25

**Authors:** Yuhang Xiao, Baojun Xu, Matteo Bordiga, Haiwei Li, Fabiano Travaglia, Shun Bai, Jiali Chen, Weibin Bai

**Affiliations:** 1 Department of Food Science and Engineering, Institute of Food Safety and Nutrition, Guangdong Engineering Technology Center of Food Safety Molecular Rapid Detection, Jinan University, Guangzhou 510632, China; xiaoyh2020@stu2020.jnu.edu.cn (Y.X.); lihaiwei720@163.com (H.L.); 2 Food Science and Technology Program, BNU-HKBU United International College, Zhuhai 519087, China; baojunxu@uic.edu.cn; 3 Dipartimento di Scienze del Farmaco, Università degli Studi del Piemonte Orientale “A. Avogadro”, Largo Donegani 2, 28100 Novara, Italy; matteo.bordiga@uniupo.it (M.B.); fabiano.travaglia@uniupo.it (F.T.); 4 Reproductive and Genetic Hospital, The First Affiliated Hospital of USTC, Division of Life Sciences and Medicine, University of Science and Technology of China, Hefei 230001, China; shunbai@ustc.edu.cn

**Keywords:** cyanidin-3-*O*-glucoside, fertility, ulcerative colitis, blood–testis barrier, inflammation

## Abstract

Impaired fertility and low sperm quality are the global health problem with high attention. It has been noted that inflammation may impact fertility by affecting testicular spermatogenesis. Cyanidin-3-*O*-glucoside is a natural functional pigment with various health benefits. Nevertheless, studies on the mechanism by which C3G protects male reproduction in mice with ulcerative colitis remain scarce. The purpose of this study is to illustrate the potential mechanism of C3G for improving impaired fertility caused by colitis. A DSS-induced colitis model was applied to assess the effects of sperm quality with colitis and the health benefit role of C3G. Results indicated that C3G-treated mice exhibited higher body weight, longer colon length, less crypt damage and focal inflammation infiltration. Being consistent with that, low sperm count, low testis weight, high inflammation levels and abnormal thickness of seminiferous epithelium also observed in the DSS group were significantly recovered upon C3G treatment. These findings suggested that colitis has a close link to impaired fertility. Further analysis found that C3G could significantly suppress the inflammatory mediators in serum. Results conjointly indicated that C3G might improve the impaired fertility of mice with colitis by inhibiting inflammatory cytokines through the blood–testis barrier. C3G could be a promising daily supplement for ameliorating impaired fertility caused by colitis.

## 1. Introduction

Inflammatory bowel disease (IBD) is a chronic inflammatory condition that mainly causes symptoms in gastrointestinal tract such as bloody diarrhea, abdominal pain, fecal urgency, incontinence and weight loss [1]. Ulcerative colitis (UC) and Crohn’s disease (CD) are representative diseases. The occurrence and prevalence of both diseases is related to genes, microbiota, immunity and environment [2,3,4,5]. With the rapid deterioration of environment as well as the change of lifestyle and diet, IBD is emerging as a global public health disease. Cohort studies over the past few decades have indicated a steadily increasing incidence of IBD in Asia, Africa, South America and countries outside the Western world [6]. Nowadays, more than 2 million in North America, 2.5 million in Europe and millions more worldwide suffer from colitis [7,8]. In China, as a rapidly developing industrialized country, the incidence of IBD has increased significantly [9].

Although the development of IBD could occur at any stage of a person’s life, the age of onset is mainly between 15 to 30 years old [10], which commonly affects young individuals. In most cases, IBD is usually diagnosed during the reproductive period, so both sexual health and fertility are serious concerns of many IBD patients [11]. Recent evidence indicates that UC one of the main IBD involved in this study, indeed affects male reproduction to varying degrees. In a short-term study with 5% (*w*/*v*) dextran sulfate sodium (DSS) and in a long-term study with 2.5% (*w*/*v*) DSS both were associated with increased inflammation, oxidative stress and DNA damage in testis. Subsequently, sperm count reduction and downregulation of 3β-HSD expression were observed in mice [12]. Further research found that a clinical trial involving 50 patients with ulcerative colitis from December 2019 to December 2020 showed that the sperm parameters of patients with ulcerative colitis were worse than those of healthy people [13]. Among them, the static parameters of sperm, including the total sperm number and the number of morphologically normal spermatozoa, and the dynamic parameters, such as vitality and progressive motility, are significantly lower than in the control group. Numerous relevant studies indicated that IBD and its associated physiological or psychological diseases impair sexual health, especially in men [11,14,15,16].

Colitis could lead to numerous injuries to the human body. However, research on the mechanisms of male reproduction affected by colitis remain limited. Inflammatory infiltration, anti-sperm antigen–antibody reaction and low leptin level under malnutrition were the potential pathways of the influence of male reproduction caused by colitis, which could be further investigated. For inflammatory infiltration, IBD is a typical symptom of local inflammation of the digestive tract. UC focusing on the present paper could trigger local inflammation of the colon. The damaged inflammatory cells and released inflammatory mediators may close to the testicles through the systemic circulation. Further disruption of the blood–testis barrier (BTB) and a decrease of the functional integrity may emerge [17]. Local or systemic inflammation impact fertility by affecting testicular spermatogenesis, steroidogenesis and testosterone synthesis via increased concentrations of proinflammatory cytokines such as tumor necrosis factor-α (TNF-α), interleukin-1 (IL-1) and interleukin-6 (IL-6) [13,18,19]. For anti-sperm antigen–antibody reaction, the intestinal mucosal barrier will be destroyed, and intestinal permeability will be increased during the process of IBD, which might lead to increased immunological response against antigens of gut microbiota possessing common antigenicity with human spermatozoa [20,21]. For low leptin level under malnutrition, malnutrition is a common complication in IBD patients with a prevalence between 6.1% and 69.7%, which depends on different screening standards [22,23]. Under the condition of malnutrition, the fat reserves decline therewith leptin level secreted by adipocytes decreases [24]. It is common knowledge that leptin is necessary for the stage of puberty growth and development [25]. It has been found that the pubertal delay in male patients with colitis is accompanied by the decrease in fat stores, which leads to the decrease of leptin level, which may be one of the potential mechanisms [26]. Moreover, it has been found that UC was associated with impaired fertility in male mice, including the elevation of testicular inflammation, oxidative stress, cell damage and DNA damage [12].

Anthocyanins are a group of flavonoids, which are widely distributed in colorful fruits, vegetables and grains. As one of the excellent antioxidants in the plant kingdom, the health benefits of anthocyanins, such as anti-oxidation, anti-inflammatory, anti-carcinogenic, retinal protection, hypolipidemia, anti-aging, cardiovascular system protection and gut microbiota modulation [27,28,29], cause its putative health-promoting effects, and therefore anthocyanins have been attracting increasing attention in recent years. Current studies of anthocyanins have focused on six major classes of anthocyanins: pelargonidin, peonidin, cyanidin, malvidin, petunidin and delphinidin [30]. Cyanidin-3-*O*-glucoside (C3G), which is the principal form of anthocyanin pigments in nature, has colitis alleviation [31] and male reproductive protection properties [32]. Growing body studies provide strong evidence that C3G ameliorates intestinal inflammation and restores intestinal structure and morphology so as to alleviate colitis [33,34,35,36]. Early studies from our team have shown that C3G may have the ability to mitigate reproductive toxicity caused by chemical poisons [32,37,38,39]. Nevertheless, relatively little attention has been focused on C3G protecting male reproduction in a mice model of chronic ulcerative colitis. Yet, just because of this, the present study aimed to investigate whether there are potential benefits of C3G for impaired sperm caused by colitis.

## 2. Materials and Methods

### 2.1. C3G Preparation and Purification

According to the pre-experimental optimization methods of our laboratory [32,38,40], C3G was extracted from the black soybean peels with acidic hydrous ethanol and then purified by macroporous resin (Rohmand Haas, Philadelphia, PA, United States) and medium pressure liquid chromatography (Li Sui Technology Co., Ltd., Suzhou, China) The final purity of C3G applied in this study was >95%, determined by HPLC-MS/MS (Shimadzu co., Ltd., Kyoto, Japan) [32].

### 2.2. Materials and Reagents

DSS (relative molecular mass 36–50 kDa) was purchased from MP Biomedicals (Santa Ana, CA, United States). ProcartaPlex™ immunoassay kit for the determination of cytokines IL-6, IL-10, IFN-α and IFN-γ was obtained from Invitrogen (Carlsbad, CA, USA). Hematoxylin and eosin (H&E) and periodic acid-Schiff (PAS) reagents were purchased from Servicebio Biotechnology Co., Ltd., (Wuhan, China). All other chemicals were of analytical grade.

### 2.3. Experimental Protocol in Mice

#### 2.3.1. Animals

C57BL/6N mice (male, weight 19–21 g, 42–48 days) were purchased from Charles River (Tongxiang, China). They were acclimated for 7 days before the start of the experiments in a specific pathogen-free (SPF) condition at an ambient temperature of 22 ± 1 °C and humidity of 40–70% under the natural photoperiod of 12-h light and dark cycle with standard chow and water. The experiments were conducted after approval by the Animal Care and Protection Committee of Jinan University. All the animals received animal welfare during experiments according to the guidance and recommendations of the Care and Use of Laboratory Animals. Efforts were made to minimize suffering and the number of animals used.

#### 2.3.2. Induction of UC Model and Treatment

Dextran sulfate sodium (DSS) was customarily employed for the establishment of the mice UC model [41,42]. In this study, the UC model was achieved according to the procedures of Chen et al. (2019) [43] with slight modifications. Briefly, mice were randomized to five groups: the control group (C), the DSS-treated group (D), the DSS plus low concentration C3G (DCL), the DSS plus medium concentration C3G (DCM) and the DSS plus high concentration C3G (DCH) (*n* = 8). In DSS-exposed groups, DSS was dissolved in the drinking water with a final concentration of 2% (*w*/*v*) in each individually ventilated cage. After the 7th day, all cages were changed to DDI water for two days. Meanwhile, the control group received drinking water without DSS throughout the experiment period.

**Figure 1:**
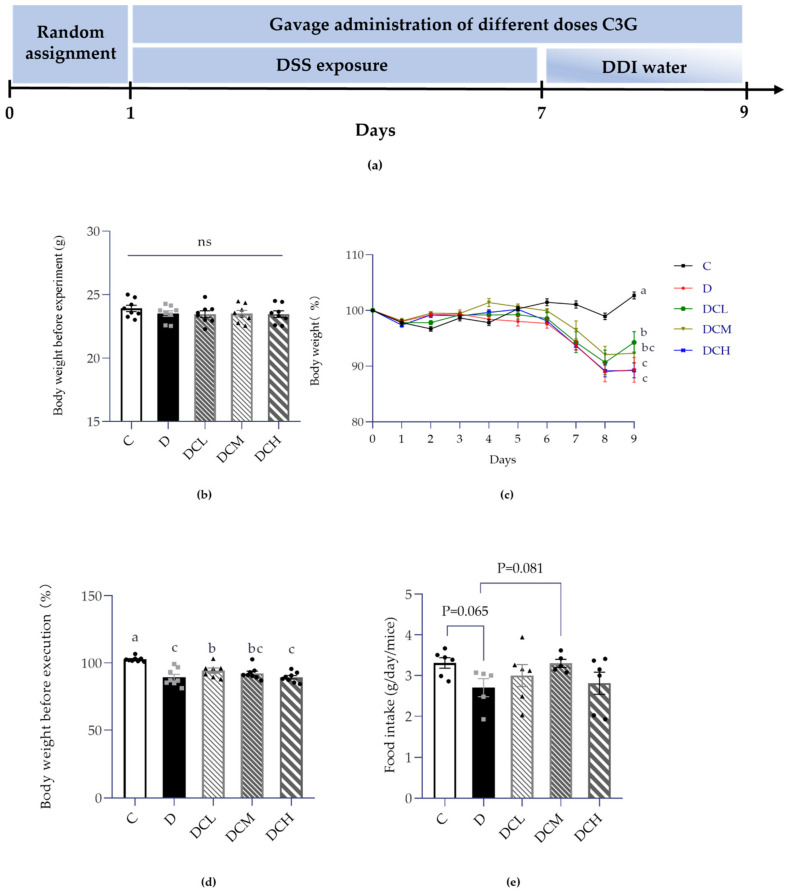
Effects of DSS on weight gain and food intake of mice (ns, non-significant; Control, C; DSS-treated mice, D; DSS-C3G (12.5 mg/kg), DCL; DSS-C3G (25 mg/kg), DCM; DSS-C3G (50 mg/kg), DCH). (**a**) Experimental timeline of UC model. (**b**) The initial weight of mice on the 0th day of the experiment. *n* = 8. (**c**) Dynamic change of body weight (shown as percentage). *n* = 8. (**d**) Body weight before execution (%). *n* = 8. (**e**) Daily food intake per mouse (g). *n* = 5~6. ▲ ● ■: The number of symbols was consistent with the number of samples, and the relative position represented the degree of data dispersion. Results were expressed as mean ± SEM. Parameters marked by the same letter are not significantly different. Significance is represented as *p* < 0.05.

During the whole experiment, low-dosage C3G (12.5 mg/kg), medium-dosage C3G (25 mg/kg), high-dosage C3G (50 mg/kg) and DDI water were daily given to mice at 0.2 mL volume by oral gavage (Figure 1a). The daily conditions of mice, comprising body weight, food intake, and defecation were observed and recorded. All mice were euthanized on the 9th day.

### 2.4. Evaluation of Histological Samples with H&E and PAS

The length of the colon (from ileocecal junction to anal verge) and the weight of testicles and caudal epididymal were measured ahead of fixed in 4% formalin over 24 h. Then, colon and testicular tissues were stained with hematoxylin and eosin (H&E) and periodic acid-Schiff (PAS), respectively. All paraffin-embedded slices were observed under Optec BK-FL microscopy (Chongqing Optec Instrument Co., Ltd., Chongqing, China) and analyzed with OPTPro software version x64 (Chongqing Optec Instrument Co., Ltd., Chongqing, China). The colon histological scoring system was referred to our previous study, followed the instruction of Chen et al. (2019) [43].

For the histopathological analysis of testis, the thickness of seminiferous tubules in testis was measured, and Johnsen’s score of spermatogenesis process was conducted for quantitative analysis. The visual fields were selected from the top, bottom, left and right of a testicular section, and 6 complete seminiferous tubules lumen were evaluated from each field, respectively. Eight mice in each group were analyzed, and each mouse had 30 complete tubules. The thickness of spermatogenic epithelium was measured from the basement membrane to the distal end of elongated sperm. The scores were ranked from 1 to 10 based on the morphology and quantity of germ cells in different stages of spermatogenesis. The detailed classification standard referred to Johnsen et al. [44]. Briefly, each seminiferous tubule was scored based on the following criteria: 1, no cells in the tubules; 2, only Sertoli cells; 3, only spermatogonia; 4, less than 5 spermatocytes; 5, many spermatocytes without spermatozoa and spermatids; 6, only 5–10 spermatids; 7, many round spermatids and/or elongated spermatids without spermatozoa; 8, only 5–10 spermatozoa; 9, Round spermatid with 120-degree acrosome but germ cells shedding or lumen obstruction with many spermatozoa; 10, the acrosome angle of round spermatid was more than 120 degrees, and there is a space in the lumen.

### 2.5. Measurement of Semen Quality

The left cauda epididymis was dissected after sacrificing the animals and minced into fraction to allow the sperm to release in phosphate-buffered saline for 5 min (pH 7.2, 37 °C). Then, samples were transferred into a pre-warmed counting chamber for further evaluation by a computer-aided semen analysis system ML-608Z11 (CASA, Song Jing Tian Lun Biotechnology Co., Ltd., Nanning, China). Twenty images were used to evaluate each mouse sample. Among them, sperm motility and static parameters of the sperm were evaluated, including mobility, vitality, sperm count and abnormality.

### 2.6. Analysis Serum of Parameters

After mice were sacrificed, whole blood was collected. Serum samples were obtained by centrifugation at 3500× *g* at 4 °C for 15 min until the supernatant has no visible red color. Subsequently, serum samples were stored at −80 °C for further analysis. A ProcartaPlex™ Immunoassay Kit from Invitrogen (Carlsbad, CA, USA) was used to analyze the serum cytokine levels according to the manufacturer’s instructions. The concentration of the samples was calculated by plotting the expected concentration of the standards against the MFI generated by each standard.

### 2.7. Statistical Analysis

Results are expressed as means ± SEM usually or medians (interquartile range) for data that are not normally distributed. Data was conducted by one-way ANOVA analysis via IBM SPSS statistical software, version 23.0 (SPSS Inc., Chicago, IL, United States). Pictures are drawn and output by GraphPad Prism V.8.0 (San Diego, CA, USA). Differences considered of statistical significance were set at *p* < 0.05.

## 3. Results

### 3.1. Effect on Weight Gain and Food Intake

DSS-induced colitis model was successfully constructed to evaluate the role of C3G in ulcerative colitis. In DSS treated group, the characteristic symptoms of UC were appeared, including weight loss, bloody stools and less fodder consumption. As shown in Figure 1b, all groups showed no differences in body weight at the beginning of the experiment. Compared to control group, mice relative body weight was significantly deceased followed by the DSS induction. Results showed that the bodyweight of mice treated with DCL and DCM achieved recovery, compared to the D group (Figure 1c,d). However, mice body weight did not show any significant changes in the DCH group, compared to D group (Figure 1c,d). Meanwhile, mice food intake also exhibited a slight improvement upon C3G (12.5 mg/kg and 25 mg/kg) treatments in comparison with the D group (Figure 1e).

### 3.2. Low Dosage C3G Ameliorated Colonic Inflammation

**Figure 2:**
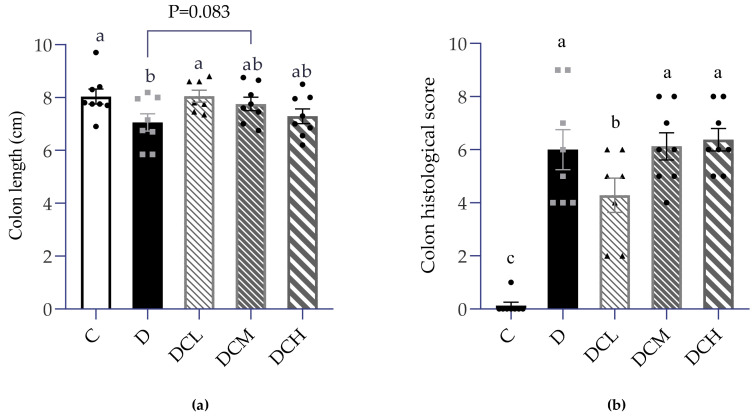

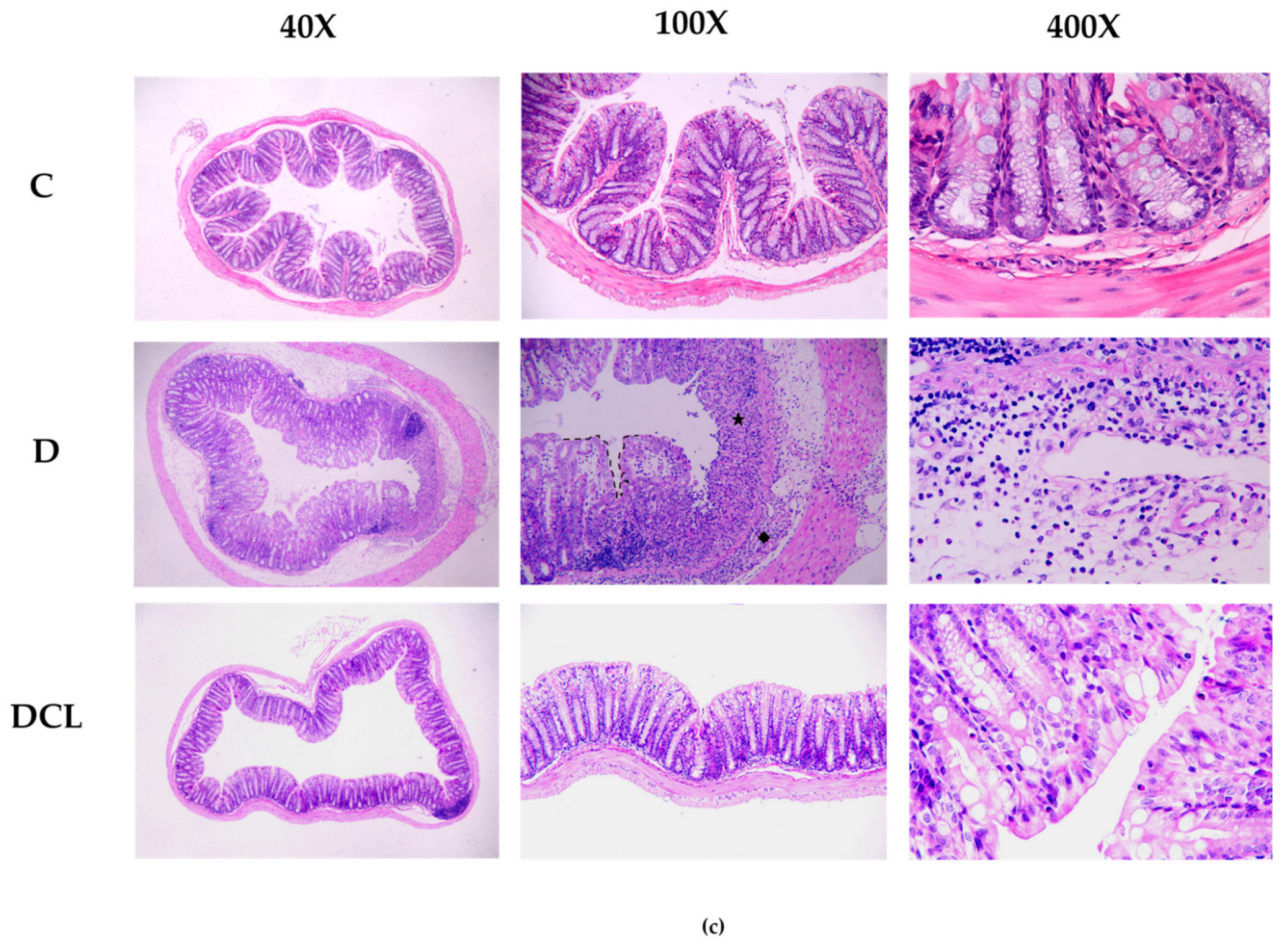
Colonic inflammation in DSS-induced colitis (Control, C; DSS-treated mice, D; DSS-C3G (12.5 mg/kg), DCL; DSS-C3G (25 mg/kg), DCM; DSS-C3G (50 mg/kg), DCH). (**a**) Determination of colon length. *n* = 7~8. (**b**) Colon histopathological changes of mice in DSS-induced colitis. *n* = 7~8. (**c**) Representative pathological pictures of H&E staining. ★: Destruction of gland structure; ◆: infiltration of inflammatory cells; ---: Changes of crypt structure: surface irregularity. ▲ ● ■: The number of symbols was consistent with the number of samples, and the relative position represented the degree of data dispersion. Results were represented as mean ± SEM. Parameters marked by the same alphabet are not significantly different. Significance is expressed as *p* < 0.05.

As previously demonstrated, mice were given 2% DSS for 7 days to induce acute colitis. As one would expect, blood clots appeared in the feces, and the stools were more watery and softer in the D group than the control group. In accordance with the results shown in Figure 1, Figure 2a also indicated that the colon of DSS-treated mice had a significantly shorter length. The DCL group exerted significant improvement in colon length *(p* < 0.05), while a slight difference appears between the DCM group and the C group *(p* < 0.1, Figure 2a). The key to the success of establishing UC was the inflammation of colon tissue, thus H&E-stained tissue sections were evaluated by combining the following indicators: severity of inflammation, the extent of inflammation, crypt damage and focal formation [43]. Score statistical analysis confirmed that inflammation condition in the D group drastically climbed up, compared to the control group. Beyond expectation, colon histopathological score in the DCL group was markedly lower than DSS-treated mice. However, the DCM and DCH had no such similar effect (Figure 2b). As shown in Figure 2c, the intact goblet cells and intestinal glands were observed but not obvious inflammatory cell infiltration, which indicated that there was no damage in the control group. Two percent DSS leads to the destruction of gland structure, infiltration of inflammatory cells in the mucosa, crypt injury and local deformation in colon tissue. However, the mice in the low-dose C3G group behaved normally, similar to the control group. Moreover, the DCL group had significantly (*p* < 0.005) lower histological scores than that examined in the D group.

### 3.3. Effects on Semen Parameters

**Figure 3:**
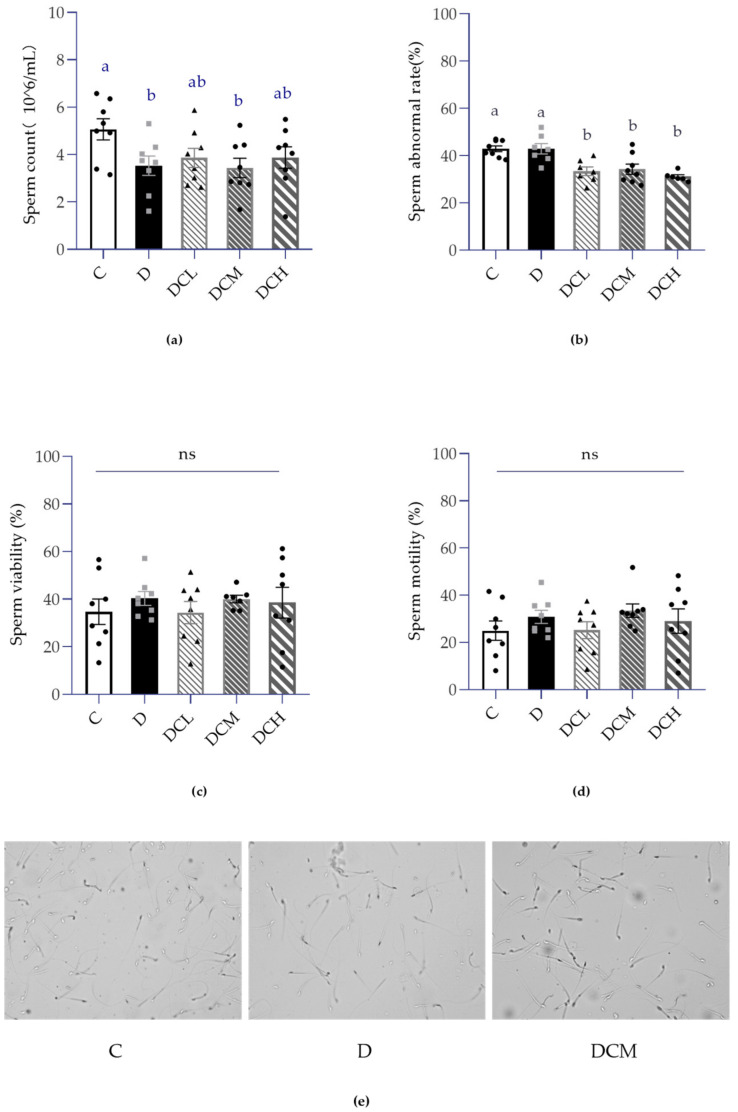
Semen parameters in mice with ulcerative colitis (ns, non-significant; Control, C; DSS-treated mice, D; DSS-C3G (12.5 mg/kg), DCL; DSS-C3G (25 mg/kg), DCM; DSS-C3G (50 mg/kg), DCH). (**a**) Sperm count per milliliter (106/mL), *n* = 8. (**b**) Sperm abnormal rate (%), *n* = 6~8. (**c**) Sperm viability (%), *n* = 8. (**d**) Sperm motility (%), *n* = 8. (**e**) Representative images of sperm capture. ▲ ● ■: The number of symbols was consistent with the number of samples, and the relative position represented the degree of data dispersion. Results were represented as mean ± SEM. Parameters marked by the same alphabet are not significantly different. Significance is expressed as *p* < 0.05.

It is a common knowledge that the sperm count, morphology and motility of sperm are the most visualized manifestations for evaluating sperm quality and male fertility. To determine the impact of UC in male reproductive biology, semen parameters were conducted to evaluate sperm production. As a whole, CASA was applied to examine sperm concentration and motility. As shown in Figure 3a, DSS-induced colitis caused a marked decrease in epididymal sperm count, while it was slightly improved upon C3G treatments. Additionally, results showed that sperm rate in the DSS-fed group was tantamount to that in controls (Figure 3b). Interestingly, administration of C3G to mice with colitis greatly diminished the mature spermatids abnormal rate in C and D groups (*p* < 0.05, Figure 3b), suggesting that C3G may protect normal spermatogenesis. Apart from mentioned parameters, sperm motility and viability were also evaluated with no significant changes (Figure 3c,d). Besides, Figure 3e shows the representative images of sperm captured by CASA, which enabled to match the trend of total sperm count and sperm abnormal rate.

### 3.4. Impaired Spermatogenesis in the DSS-Induced Mice

**Figure 4:**
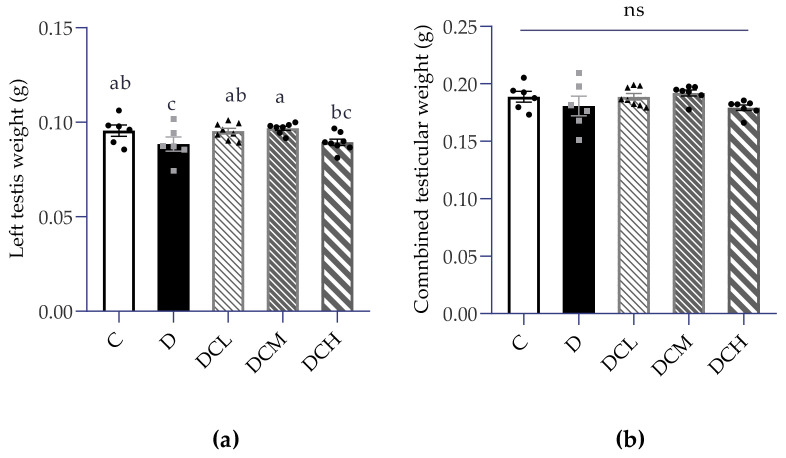
Absolute weight of male reproductive organs (ns, non-significant; Control, C; DSS-treated mice, D; DSS-C3G (12.5 mg/kg), DCL; DSS-C3G (25 mg/kg), DCM; DSS-C3G (50 mg/kg), DCH). (**a**) Left testis weight, *n* = 7–8. (**b**) Combined testicular weight, *n* = 6–8. ▲ ● ■: The number of symbols was consistent with the number of samples, and the relative position represented the degree of data dispersion. Results are shown as mean ± SEM. Parameters marked by the same alphabet are not significantly different. Significance is expressed as *p* < 0.05.

When the experiment comes to an end, mice were sacrificed and sex organs testicles were weighed separately, which gently peeled off fat tissue quickly. Testes were processed in parallel following described in the manufacturer’s instructions. Data statistics disclosed a similar combined testicular weight between groups (Figure 4b), but surprisingly, there was an evident inclination of the decreased absolute weight of left testis in the D group (Figure 4a). Meanwhile, we observed that DCL and DCH groups restored the weight of left testis to the control level.

**Figure 5:**
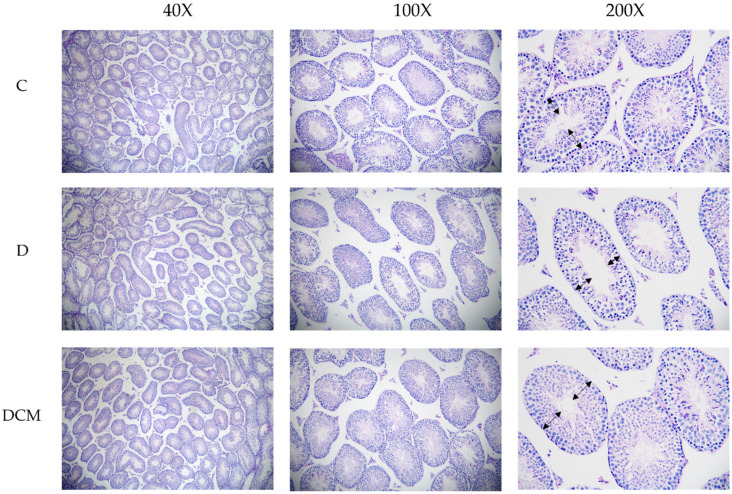
Histopathological analysis of C3G on PAS-stained testis of mice in DSS-induced colitis. ←→: Thickness of spermatogenic epithelium.

**Figure 6:**
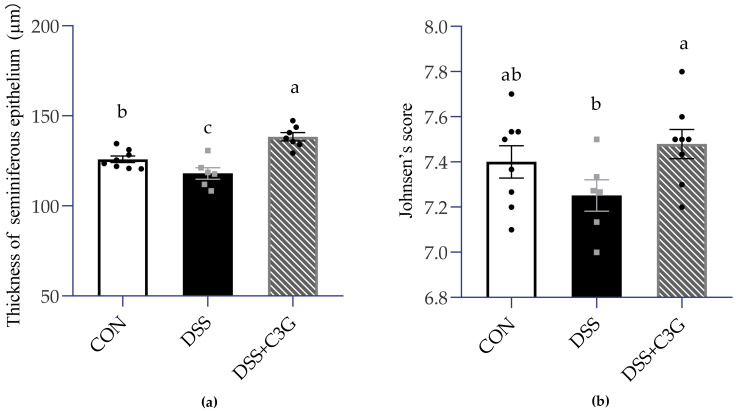
Impaired spermatogenesis in the DSS-induced mice. (**a**) Thickness of spermatogenic epithelium (μm). The thickness is obtained by subtracting the inner diameter from the outer diameter, *n* = 6–8. (**b**) Johnsen’s score of PAS-stained testis. ● ■: The number of symbols was consistent with the number of samples, and the relative position represented the degree of data dispersion. Results were represented as mean ± SEM. Parameters marked by the same alphabet are not significantly different. Significance is expressed as *p* < 0.05.

Furthermore, a quantitative analysis of testis was proceeded, including the measurement of spermatogenic epithelium thickness and Johnsen’s score in control group (C), DSS-treated group (D) and intervention of medium-dose C3G group (DCM) to confirm the influence of UC associated inflammation on seminiferous tubule in testis. Based on the histomorphometric analysis, there was a remarkable significance between the three groups in terms of thickness of seminiferous tubule (Figure 5 and Figure 6). Thinner seminiferous tubules, which means that the number of spermatogenic cells is relatively less, were observed in Group D (Figure 5). Johnsen’s score is an effective method to quantify spermatogenesis. The animals in the model (DSS-received) group represented a slight (*p* = 0.156) decrease in Johnsen’s score (7.25 ± 0.07) compared to the control (7.40 ± 0.07). The DCM group showed a significant (*p* < 0.05) increase in Johnsen’s score (7.47 ± 0.06) compared to the D group (Figure 6b). These results indicated that UC had a negative effect on the histomorphology of seminiferous tubules.

### 3.5. Expression of Inflammatory Factors in Serum

**Figure 7:**
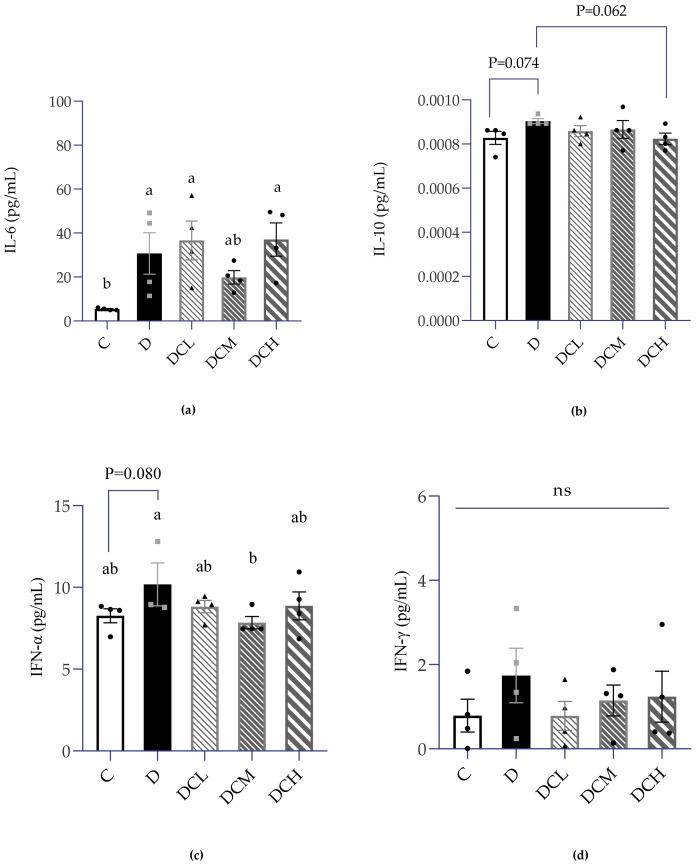
Expression of inflammatory factors in serum. (**a**) IL-6, *n* = 8. (**b**) IL-10, *n* = 8. (**c**) IFN-α, *n* = 6–8. (**d**) IFN-γ, *n* = 8. In this figure, a dot represents two mice. Results were expressed as mean ± SEM. ▲ ● ■: The number of symbols was consistent with the number of samples, and the relative position represented the degree of data dispersion. Parameters marked by the same alphabet are not significantly different. Significance is expressed as *p* < 0.05.

Inflammatory factors play a vital role in the development of IBD. Therefore, a series of inflammatory factors in serum were analyzed, comprising IL-6, IL-10, IFN-α and IFN-γ. As shown in Figure 7a, compared to the control group (5.378 ± 0.25 pg/mL), the serum concentration of IL-6 in the DSS model group (30.71 ± 18.88 pg/mL) was strikingly higher (∼6-fold). The level of IL-6 could be pulled back by supplementation with a medium dose of C3G to a level that is not significantly different from that of the control group. However, the serum interleukin-10 of UC mice was only slightly different from that of normal mice (*p* = 0.074, Figure 7b). The serum IFN-α increased slightly in the D group (*p* = 0.080), but it decreased to a normal level in the DCM group (Figure 7c). The experimental data provide evidence that serum IFN-γ (Figure 7d) have no change between groups.

## 4. Discussion

**Figure 8:**
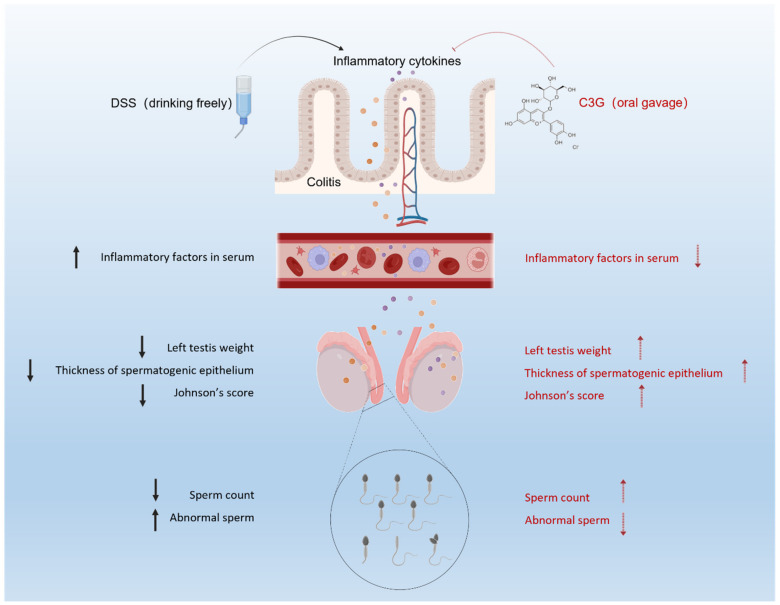
Schematic diagram of C3G improves the impaired fertility of mice with colitis by inhibiting inflammatory cytokines through the blood–testis barrier.

The purpose of this study was to investigate whether C3G mitigates male reproductive damage with colitis. DSS-induced colitis is a frequently used model to construct ulcerative colitis in mice [45]. Similar to previous studies, the primary features of ulcerative colitis were observed, such as weight loss, reduction of food intake, shortening of colon length, aggravation of intestinal inflammation, goblet cell loss, bloody diarrhea and loose stool, indicated that the colitis model was successfully constructed [46,47,48]. Current results clearly showed that C3G at the dose of 12.5 mg/kg (DCL) and 25 mg/kg (DCM) were the effective concentrations for improving impaired fertility and colitis amelioration. A recent report found that a low dose of cyanidin-3-*O*-glucoside could alleviate DSS-induced colitis by mediating the CD169+ macrophage pathway [34]. Previous studies also indicated that C3G plays a critical role during spermatogenesis [38]. Consistent with that, present results demonstrated that dietary C3G supplements possessed health benefits on UC and male reproductive damage (Figure 8).

Notably, the reductions of body weight (*p* < 0.05, Figure 1c,d) and food intake (*p* = 0.065, Figure 1e) suggested the severity of ulcerative colitis in DSS-fed mice. The consequence might be caused by less food intake, food indigestion as well as nutrient malabsorption in the gastrointestinal injury of colitis [49,50]. Malnutrition would aggravate inflammation and further worsen the physical condition [51].

The testicular weight difference is a sensitive indicator in male reproduction, especially in toxicological experiments, and must be observed. UC is usually accompanied by weight loss, yet testicular weight may also decrease due to the special effects of toxicity. Herein, in the case of no obvious change of organ coefficients, the absolute weight of testis is a key index that is more sensitive than fertility experiment [12]. Simultaneously, the weight of testis in UC group decreased significantly, which may be due to the reduction in the thickness of spermatogenic epithelium or the number of spermatogenic cells. Current results found that left testis weight in D group (Figure 4a) was significantly lower in comparison to control group, demonstrating the appearance of impaired fertility. Further investigation indicated that the D group exhibited significantly lower sperm count and higher sperm abnormal rate, confirming that impaired fertility and low sperm quality was accomplished with colitis (Figure 3a,b). Similar to the previous report that C3G possessed health benefits in improving sperm quality, the abnormality of above mentioned sperm parameters caused by colitis were obtained a recovery upon C3G treatments [12].

Just as mentioned above, the sperm count and left testis weight decreased obviously in the DSS-fed group, and oral administration of C3G had an effective therapeutic effect. Further evaluation of spermatogenesis in testis was necessary. As expected, the thickness of seminiferous tubules in male mice with UC was obviously smaller than that in the normal group, which might be due to the decreased number of spermatogenic cells. Further considering the morphological state of and numbers of germ cells at different stages of spermatogenesis [52], we chose Johnsen’s score, which is a histological quantitative scoring system with the advantages of reliability and easy application [44,53]. Johnsen’s score is also a histopathological index that is easy to predict male reproductive ability and commonly used to predict semen quality of male infertility patients after receiving various treatment indications [54]. The lowest score appeared in the colitis mice model, which was coincident with the data provided by Chang et al. [55]. This was related to the irregular spermatogenesis cycle and absence of sperm and spermatocytes, which increased the possibility of sperm damage.

Colon length and histopathological score are highly correlated with inflammatory activity [56]. When low-dose C3G was used to intervene, the colon length could be best rectified compared with high and middle doses (Figure 2a). Microscopic observation and scoring of H&E stained colon sections showed a large number of inflammatory cells including neutrophils, monocytes and lymphocytes in the lamina propria and submucosa of mucosa in the model group. Consistent with the above results, the pathological score of 12.5 mg/kg C3G group was significantly lower, and colon morphology was closer to that of the control group compared with those in the DSS group (Figure 2b,c).

Inflammation is one of the main mechanisms of the occurrence and progress of UC. Besides, inflammatory cells or inflammatory factors reach all parts of the body through systemic circulation, which may induce other diseases related to the internal environment. Some clinical and pre-clinical studies also demonstrated that ulcerative colitis could lead to a significant increase in serum inflammatory factors, such as IL-17A, IL-6 and CRP [57,58]. Consistent with earlier research, we found that serum IL-6 levels were significantly elevated compared to those in control mice. The concentration of IL-6 is positively correlated with the degree of inflammation and has been proved that it can stimulate or even aggravate UC by affecting the normal function of intestinal epithelial cells [59]. Emerging studies have demonstrated that the proinflammatory state in males is also associated with an impaired reproductive system [60,61]. Animal experiments show that chronic inflammation can affect spermatogenesis, sperm quantity, and quality, which may be caused by inflammation and oxidative stress related to UC [12]. As mentioned earlier, in the UC mice, the persistent inflammatory infiltration of colon may contact the testicles through blood circulation, which has a negative impact on the testicles. Inflammatory factors may further destroy the blood–testis barrier and enter the apical compartment of seminiferous tubules to affect spermatogenesis [17]. A recent study indicated that the increase of serum TNF-α and IL-6 levels was accompanied by the increase of mRNA expression of TNF-α, IL-1β and IL-6 in testis [62], which indicated that the overexpression of inflammatory factors in serum might have adverse effects on the male reproductive system. In agreement with previous studies, we found that 25 mg/kg C3G significantly reduced the levels of TNF-α and IL-6 in serum, suggesting that low dose C3G may attenuate systemic and testicular inflammatory responses. Therefore, a relatively low-dose C3G (12.5 mg/kg and 25 mg/kg) may alleviate the damage of inflammatory factors to testis by improving colitis and reducing the level of serum inflammatory factors. Colitis may disrupt the blood-testis barrier and then affect sperm quality and impaired fertility. The present study illustrated the therapeutic potential of C3G in improving sperm quality and fertility of mice with colitis. A further justification for the target pathways investigation of C3G in protecting male reproduction to avert fertility impairment from colitis is necessary.

## 5. Conclusions

Taken together, jointly results elucidated the potential mechanism of C3G protects male reproduction in mice with ulcerative colitis. DSS-induced colitis model was applied in this study to assess the effects of sperm quality with colitis, and the health benefit role of C3G. Results indicated that C3G-treated mice exhibited higher body weight, longer colon length, less crypt damage and less focal inflammation infiltration in comparison with DSS model group, confirmed that C3G supplement could ameliorate colitis. Being consistent with the results from colitis amelioration, low sperm count, low testis weight, high inflammatory levels in serum and colonic tissue, and abnormal thickness of seminiferous epithelium were observed in DSS group that were significantly recovered upon C3G treatment, especially for low dosage (12.5 mg/kg) supplement. These findings suggest that colitis has a close link to low sperm quality and impaired fertility. Further analysis found that C3G supplements could significantly suppress the inflammatory mediators in serum. Current results conjointly indicated that C3G could improve the sperm quality and impaired fertility of mice with colitis by inhibiting inflammatory cytokines through the blood–testis barrier. The present findings confirmed the therapeutic potential of C3G in ameliorating ulcerative colitis and provided a potential pharmacological basis for further application of C3G in ameliorating impaired fertility caused by colitis.

## Data Availability

Data will be available at request.
